# Simultaneous Droplet Generation with In-Series Droplet T-Junctions Induced by Gravity-Induced Flow

**DOI:** 10.3390/mi12101211

**Published:** 2021-10-04

**Authors:** Khashayar R. Bajgiran, Alejandro S. Cordova, Riad Elkhanoufi, James A. Dorman, Adam T. Melvin

**Affiliations:** Cain Department of Chemical Engineering, Louisiana State University, Baton Rouge, LA 70803, USA; kramez1@lsu.edu (K.R.B.); acordo7@lsu.edu (A.S.C.); riad.elkhanoufi@gmail.com (R.E.)

**Keywords:** T-junction droplet generator, gravity-driven flow, multiplexing, droplet tuning

## Abstract

Droplet microfluidics offers a wide range of applications, including high-throughput drug screening and single-cell DNA amplification. However, these platforms are often limited to single-input conditions that prevent them from analyzing multiple input parameters (e.g., combined cellular treatments) in a single experiment. Droplet multiplexing will result in higher overall throughput, lowering cost of fabrication, and cutting down the hands-on time in number of applications such as single-cell analysis. Additionally, while lab-on-a-chip fabrication costs have decreased in recent years, the syringe pumps required for generating droplets of uniform shape and size remain cost-prohibitive for researchers interested in utilizing droplet microfluidics. This work investigates the potential of simultaneously generating droplets from a series of three in-line T-junctions utilizing gravity-driven flow to produce consistent, well-defined droplets. Implementing reservoirs with equal heights produced inconsistent flow rates that increased as a function of the distance between the aqueous inlets and the oil inlet. Optimizing the three reservoir heights identified that taller reservoirs were needed for aqueous inlets closer to the oil inlet. Studying the relationship between the ratio of oil-to-water flow rates (Φ) found that increasing Φ resulted in smaller droplets and an enhanced droplet generation rate. An ANOVA was performed on droplet diameter to confirm no significant difference in droplet size from the three different aqueous inlets. The work described here offers an alternative approach to multiplexed droplet microfluidic devices allowing for the high-throughput interrogation of three sample conditions in a single device. It also has provided an alternative method to induce droplet formation that does not require multiple syringe pumps.

## 1. Introduction

Droplet microfluidic devices have been demonstrated for a broad range of applications including nanoparticle synthesis [1,2], chemical reactions [3], protein crystallization [4], biological assays, and cellular analysis [5,6,7,8]. In most instances, uniform droplets are desired to ensure constant, controlled, and predictable outcomes. Conversely, some applications require a wide range of tunable droplet volumes, typically femtoliters to nanolitres. Therefore, it is critical to have a deep and systematic understanding of microfluidic droplet formation. Microfluidic droplet production has been primarily achieved through T-junctions [9] or flow-focusing junctions [10]. The commonly used flow-focusing generators can deliver monodisperse droplets at low capillary numbers (Ca) when droplets are produced in a highly stable breakup process (e.g., dripping regime) [11]; however, a complex velocity field and several key parameters defining the geometry (e.g., oil and water channel width and height) have made it challenging to model flow-focusing geometries analytically [12]. The T-junction is a widely applied geometry for droplet generation due to the ease in droplet-size controllability and consistency of drop formation and design simplicity [13]. Despite the plethora of studies on simultaneous emulsification in multi-input flow-focusing microfluidic devices [14,15,16], the reports on integrated in-series T-junction droplet generators have been limited. The challenge in the multiplexed production of droplets is to prevent the broadening of droplet size distribution due to complications involved with simultaneously using multiple droplet generators, which is more evident in series T-junction droplet generators [17].

Another challenge with multiple droplet generators is the hardware requirement for droplet generation is often cost-prohibitive [18]. Syringe pumps are most commonly used to generate a constant flow from the external macro-environment into a micro-channel; however, when a syringe pump is applied to a micro-channel, flow fluctuations can occur due to the motion of the electric motor and deformation of elastic channel walls [19,20]. Additionally, other technical limitations, such as slow fluid response time, low volume dispensed, and unwanted pulsatile fluid flow, lead to chip leakage and device de-bonding [18]. Microfluidic chips that use multiple inlets often require >3 syringe pumps, which can be costly and complicated to operate. Alternatively, pressure pumps can be used to induce flow more stably with faster responses than those of the syringe pumps; however, accurate control of fluid flow with the pressure source is difficult to achieve in the presence of parametric uncertainties or disturbances in a microfluidic network [21]. For example, fabrication error, air bubbles in the channel, the wobble of tubes, suspended cells, or the swelling of the channel can inhibit the accuracy of flow regulation [22]. To overcome these limitations, a number of low-cost, zero electric power consumption, and portable on-chip passive micropumps have been developed [23,24]. Among these pumping mechanisms, the gravity-driven flow is the most straightforward and commonly used method in various microfluidics-based applications [25]. Gravity-driven flow requires liquid reservoirs with different heights to achieve fluid propulsion from the higher reservoir to the lower one [26,27,28]. A key advantage of gravity-driven flow is that the inlet liquid can be adjusted in real time to monitor different conditions and prolong flow. Furthermore, it is naturally a low maintenance type of flow, requiring no moving mechanical parts [29]. Using gravity flow solves the notorious air bubble formation problem, since air bubbles are prevented from entering the device due to their buoyancy, eliminating the need for external or in-line bubble traps [30,31]. To date, the majority of gravity-driven platforms have been limited to single-input designs, with very few studies examining their potential in multiple droplet generators on a single device [32,33].

In this work, a multi-aqueous-input, in-series T-junction microfluidic droplet device was developed that leverages easy-to-use gravity-driven flow control. The rationale behind this device design was twofold: first, incorporating three droplet generators on a single device enables multi-condition evaluation of relevant parameters all in a single run which can save time, reagents, and labor in applications such as personized medicine [34]. This is particularly critical when working with primary samples and patient biopsies as they are oftentimes limited with sample volume and duration for subculturing. Second, the T-junctions were positioned in-series to decrease the overall size of the microfluidic chip compared to a parallel design. The in-series droplet microfluidic device incorporated four inlet ports: one oil inlet and three aqueous inlets, each containing three different inlet fluid compositions. The gravity-induced flow was incorporated to determine if the in-series T-junctions could yield uniform size droplets with comparable droplet generation rates. Prior to droplet generation, each aqueous inlet reservoir’s height was optimized through the development of an empirical model. The model is dependent on a series of fluid flow rate measurements from multiple combinations of “active” inlets (e.g., alternating the number and position of the inlets with aqueous flow inside the device) in the absence of oil flow to characterize the relationship between reservoir height and flow rate in the device. Using the optimized inlet reservoir heights, a series of experiments were performed using double active inlets to identify the allowable oil to water ratio (the volumetric oil to water flow rate, Φ) and its effect on the droplet size. Decreasing this ratio resulted in increasing droplet diameters for all inlet combinations, allowing for facile droplet size tuning.

## 2. Materials and Methods

**Figure 1:**
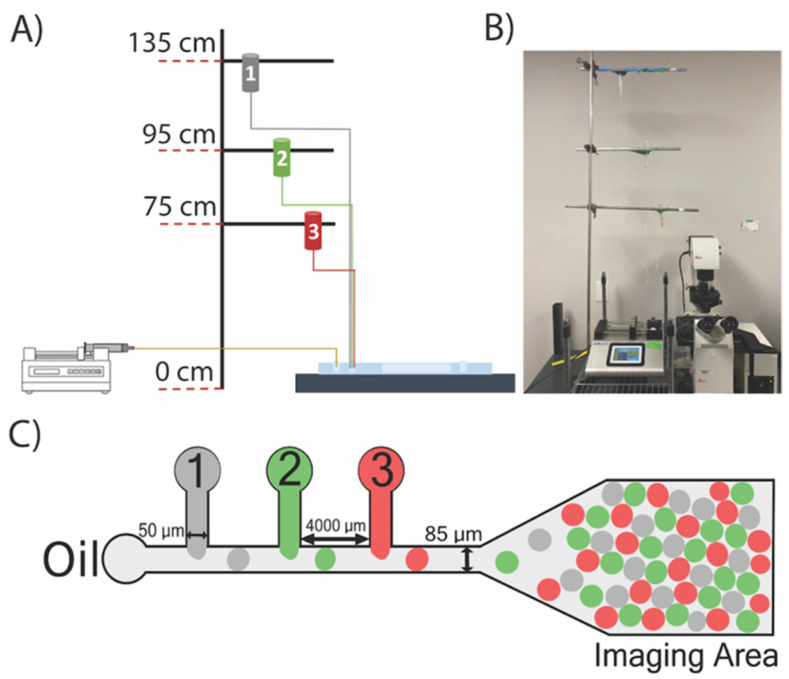
**Microfluidic Device Design and Operation.** (**A**) Schematic of the developed gravity-driven platform where reservoirs at different heights were used to initiate the flow in the aqueous inlets. A syringe pump was used for oil inlet; (**B**) photograph of the developed droplet microfluidics platform including the gravity flow/syringe pumps, and the device mounted on the Leica Microscope; (**C**) the schematic of the droplet microfluidics system showing different parts of the device during a TA experiment.

The droplet microfluidic device consisted of three in-series T-junction droplet generators, each spaced 4000 µm apart (Figure 1). The channel width for the aqueous and oil inlets was set to 50 and 85 µm, respectively. The microfluidic devices were fabricated by a combination of soft lithography and PDMS replication [8]. The geometry was designed in AutoCAD (Autodesk, Mill Valley, CA, USA) to generate a transparency mask (CAD/Art) of the fluidic channels. Two-step soft-lithography was used to fabricate the silicon master. A 40 μm-thick negative photoresist polymer (SU-8 2025, Kayaku Advanced Materials, Inc., Westborough, MA, USA) was deposited on a clean 3” silicon wafer (University Wafer, South Boston, MA, USA) using a spin coater (WS-650MZ-23NPP, Laurell Technologies, San Diego, CA, USA) and baked at 65 °C for 10 min followed by a second bake at 95 °C for 20 min. After the wafer was cooled to room temperature, the transparency mask was placed on top of the wafer, followed by exposure to UV light (1.4 mW cm^−2^) for 45 s in a custom-built UV exposure set-up using a B100-AP lamp (VWR, Radnor, PA, USA). The wafer was baked again at 65 °C for 15 min and 95 °C for 30 min. The silicon wafer was developed with a SU-8 developer solution (Kayaku Advanced Materials, Inc.), removing the uncrosslinked SU-8 to produce the microfluidic patterns. The wafer was hard-baked at 150 °C for 30 min to increase wafer durability.

PDMS replicas (Slygard 184, Ellsworth Adhesives, Germantown, WI, USA) were generated by mixing the base agent in a 10:1 ratio with the curing agent, followed by degassing in a vacuum chamber to create a bubble-free mixture. This PDMS was poured on the silicon master and was cured for at least 6 h at 65 °C. Once cured, the PDMS was removed from the wafer, and the inlet and outlet ports were punched using a blunted 18-gauge needle. The PDMS replicas were permanently bonded to 25 mm × 75 mm glass slides (Corning Inc., Corning, NY, USA) using an O_2_ Harrick Plasma PDC-32G basic plasma cleaner with a 30 s exposure to plasma. The devices were left overnight to ensure proper bonding between the PDMS and the glass. The fluidic channels in the microfluidic device were made hydrophobic by manually injecting Aquapel (PGW Auto Glass, LLC, Cranberry Township, PA, USA), using a filtered syringe with excess Aquapel, and flushed with Novec 7500 oil (3M, Saint Paul, MN, USA) to ensure proper droplet formation. Finally, the channels were dried by blowing nitrogen.

## 3. Results and Discussion

### 3.1. Non-Uniform Reservoir Heights Were Required to Generate Uniform Droplets Using Gravity-Driven Flow

**Figure 2:**
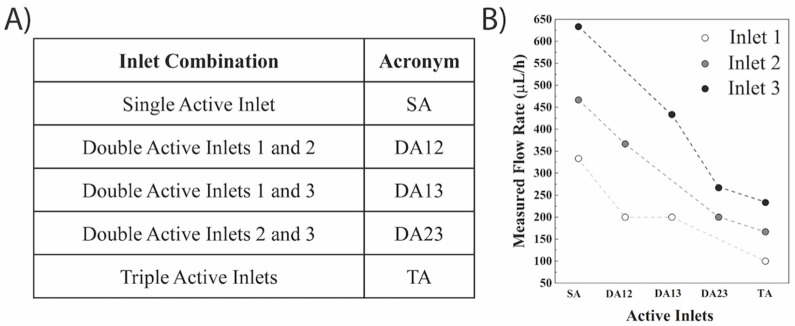
**Summary of the results of the gravity-driven flow height optimization in the absence of oil flow.** (**A**) Table summarizing the different active inlet conditions and their respective acronyms; (**B**) plots of single active inlet (SA) experiments have been compiled into the first column. Similarly, each double active inlet (DA) experiment has been compiled into a column and tagged with the respective active inlets. Finally triple active inlets (TA) experiment has been shown in the last column.

An essential aspect of droplet microfluidic is to generate droplets of uniform size to ensure accurate comparisons between the cargo inside the droplet. Droplets of uniform size can be easily tuned by adjusting the ratio between the oil and aqueous flow rates. As such, the first set of experiments involved measuring the flow rate from a single active aqueous inlet in the absence of oil flow (Figure S1). Flow rates of 333 µL/h, 467 µL/h, and 633 µL/h were observed for single active aqueous inlets 1, 2, and 3, respectively (Figure 2). The difference between the flow rates can be explained in part by the higher flow resistance experienced by the inlets closer to the oil inlet from the channel walls since the fluid from these inlets traveled through the narrow aqueous and oil channels for longer [35]. This difference can also be explained by the proximity of the active aqueous inlet to the widening of the channel downstream of the T-junctions in accordance with the Hagen-Poiseuille equation (see Supporting Information) [18]. The presence of the widened downstream channel could result in a pressure drop under low Reynolds number flow (Re = 1.5, 2.1, and 2.8 for inlets 1, 2, and 3, respectively), which could be modeled by the Hagen-Poiseuille law using Equation (S1). Assuming that the pressure changes were negligible for an outlet channel width higher than 1500 µm [36], the difference in pressure drop from the aqueous inlets to the widened fluidic channel resulted in an increase in the flow rate from the aqueous inlets. This confirmed that uniform reservoir heights were not suitable to generate similar aqueous inlet flow rates.

A major challenge in simultaneous droplet generation is the parametric coupling of the droplet generators where the formation of droplets at a particular junction affects droplet formation at another one [17,37]. This necessitates control and balance between the pressure and flow rate over the entire set of generators. As such, the use of multiple active aqueous inlets was first investigated to study the interaction between the presence of one, two, or three active inlets at the same height (125 cm). Three combinations of two active aqueous inlets were studied (Figure 2A) to elucidate the effect of multiple in-line T-junctions in the absence of oil flow (Figure S2). The different combinations of active aqueous inlets resulted in a range of flow rates with a general trend that the presence of a second active aqueous inlet resulted in a net decrease in the flow rate for both aqueous inlets (Figure 2B). This can be explained by the hydrodynamic resistance exerted on a given aqueous inlet from another active inlet [17]. It was found that when the active aqueous inlets were adjacent to each other (e.g., DA12 or DA23), the parametric coupling between them decreased both inlets’ flow rate more drastically compared to when the inlets were further apart (Figure 2B). For instance, the flow rate of inlet 3 was 433.3 µL/h in DA13 compared to 266.7 µL/h for DA23. It was also observed that inlet 1, the closest to the oil inlet, was only able to achieve a max flow rate of 200 µL/h when coupling with a second active inlet. A single combination of all three active aqueous inlets (TA) was implemented in the absence of oil flow with a fixed height of 125 cm for all three reservoirs (Figure S3). This resulted in a further overall decrease in the flow rates for all three inlets of 100, 167, and 233 µL/h for inlets 1, 2, and 3, respectively. It should also be noted that since each reservoir contained 12 mL of overhead liquid, and considering the slow flowrate inside the microfluidic device, the amount of liquid used even after 2–3 h of experimentation was ~0.3–0.5 mL. This slight decrease in liquid height was found not to significantly affect the flow rate, droplet size, and generation rate.

An empirical model was developed for the aqueous flow-only experiments to characterize the system and predicted the relationship between reservoir height and aqueous flow rate (see Supporting Information). Using the data from single, double, and triple active inlets at fixed reservoir height (125 cm), the constants (A_xy_) in Equations (S2)–(S4) were determined (Table 1). Setting the aqueous inlet reservoirs at 125 cm (inlet 1), 95 cm (inlet 2), and 75 cm (inlet 3) yielded similar measured flow rates (148–152 µL/h) to ensure monodisperse droplets from all inlets (Table 1). The predicted flow rates were compared to measured flow rates with a relatively low coefficient of variances (CV, 8.4–12.9%). The slight difference between predicted and measured flow rates are similar to experimental reproducibility variations observed by Liang et al. when characterizing a degas-driven flow in a microfluidic device [24]. The higher measured flow rate of inlet 1 was set to overcome the backpressure imposed by the oil inlet. To test the model accuracy, the height of each inlet reservoir was calculated using the model so that all inlets yield flow rates of 100 μL/h. The revised reservoir heights were set to 75 cm (inlet 1), 55 cm (inlet 2), and 47 cm (inlet 3), and all cases resulted in a measured flow rate close to 100 μL/h with low coefficient of variances (CV, Table S1).

### 3.2. Decreasing the Oil-to-Water Ratio (Φ) Increased Droplet Diameter without Affecting Droplet Uniformity

Recent studies in T-junction designs have investigated the impact of the flow characteristics, such as flow rates [38], liquid properties [39], and channel dimensions [40] on the droplet size. The oil-to-aqueous flow rate ratio (Φ) has been suggested as a prominent parameter governing droplet size in the squeezing regime [41]. Varying Φ is the simplest way to control droplet size, which is why it has been chosen as the key contributing factor in controlling the droplet generation here. It should be noted that changes in the oil phase velocity in this system, which result in the changes in oil-to-water flow rate ratio, can be directly converted in changing the *Ca* number using the equation below:

$$Ca=\frac{\mu U}{\gamma }$$

where *μ* is the continuous (oil) phase viscosity, *U* is the oil phase velocity, and *γ* is surface tension. Thus capillary number can be easily used as the independent parameter instead of the oil-to-water flow rate ratio affecting the droplet size/generation rate.

**Figure 3:**
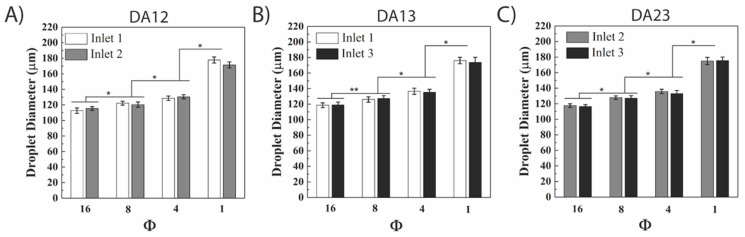
Droplet diameter increased as the oil-to-water ratio (Φ) value decreased in double active gravity-driven droplet generation. The relationship between Φ and droplet diameter was elucidated using active inlets 1 and 2 (**A**), inlets 1 and 3 (**B**), inlets 2 and 3 (**C**). The mean droplet diameter from the three different inlets at the same oil-to-water ratio were not significantly different from each other at a given Φ for each experiment. However, the cumulative inlet averages across different Φ values were statistically different with * denoting *p* > 0.05, ** denoting *p* > 0.01 thresholds. A minimum of 30 droplets were analyzed for each condition.

A series of two active aqueous inlet experiments were performed to identify the Φ range in the microfluidic device. The inlet reservoirs were set to the optimized heights described in the previous section (Table 1) to deliver equal aqueous flow rates, while the oil inlet flow rate was varied between 30–240 µL/h resulting in a range of Φ between 1–16. The droplet diameters observed from each of the active inlets for all Φ values using the three DA combinations (DA12, DA13, and DA23) were monodisperse with no statistical significance observed using a one-way ANOVA (Figure 3). For instance, at Φ = 8, the average droplet diameters for inlets 1 and 3 were 126.1 ± 3.5 µm and 127.4 ± 3.6 µm in the DA13 combination. Similarly, at the same Φ, the average droplet diameter for inlets 2 and 3 were 127.9 ± 2.3 µm and 127.1 ± 3.2 µm in DA23 combination. However, statistically different droplet diameters were observed when comparing different Φ values. In all DA combinations, the average droplet diameter was inversely proportional to Φ. For example, in DA12, decreasing Φ from 16 to 4 to 1 resulted in an increase in average diameter from 114.1 ± 3.1 to 129.6 ± 2.7 µm to 174.7 ± 3.9 µm. This dramatic shift could be explained by the change in the flow regime from dripping to squeezing [11]. When the average droplet diameters were compared across all Φ values using Fisher’s LSD test, it was found that all DA combinations resulted in significantly different values (Figure 3A–C, Tables S2–S4). This verified the system’s capability of achieving tunable droplet diameters by modifying the oil flow rate.

**Figure 4:**
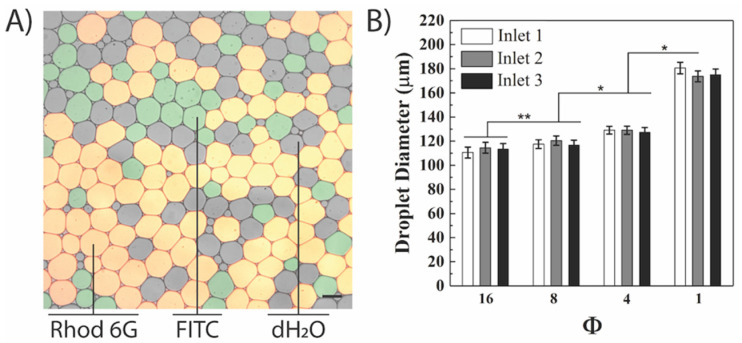
**Decreasing the oil-to-water ratio (Φ) increased droplet diameter in gravity-driven flow.** (**A**) Overlay fluorescence microscopy image of the droplets generated from inlets 1 (dH_2_O), 2 (FITC), and 3 (Rhodamine 6G) at Φ = 16. Scale bar represents 100 μm; (**B**) the mean droplet diameter from the three different inlets at the same oil-to-water ratio were not significantly different from each other at a given Φ. However, the cumulative inlet averages across different Φ values were statistically different with * denoting *p* > 0.05, ** denoting *p* > 0.01 thresholds. For each inlet, 30 droplets were measured, and the results are representative of a single trial.

The next step was studying the relationship between droplet diameter and the same four Φ values using three active aqueous inlets (Figure 4). An overlay of the three microscopy images (two fluorescent channels and a brightfield channel) allowed for facile identification of the droplets generated from the three aqueous inlets (Figure 4A). Similar droplet diameters were observed in droplets generated from each aqueous inlet for all four Φ values (Figure 4B). For example, at Φ = 16, the average droplet diameter for inlets 1, 2, and 3 were 110.5 ± 4.6, 114.5 ± 4.5, and 113.5 ± 4.4 µm, respectively. Similar to DA experiments, droplet diameters were found to have an inversely proportional relationship to Φ (Figure 4B), supported by previous reports for a single T-junction [42]. This finding can be explained by looking at the fluid dynamics governing a T-junction. As the flow rate of the continuous oil phase (and therefore its velocity) decreased (with decreasing Φ), larger aqueous droplets formed due to the decrease in shear stress acting on the emerging aqueous filament at the T-junction. Conversely, reducing the flow rate of the dispersed aqueous phase resulted in smaller droplets since the emerging droplet tip was “deflated” with smaller amounts of the dispersed phase liquid. One finding observed across all experiments was that <5% of the droplets generated (which were not included in the analysis) were either too small (<70 μm) or too large (>300 μm), which can be explained by droplet breakups that occur when the droplets enter the main channel and encounter PDMS residue. In the TA combination, the increase in droplet diameter was observed at Φ > 8; however, values of Φ lower than 8 (specially Φ = 1) resulted in a statistically significant change in droplet diameters with a higher level of significance (0.01 versus 0.05, Figure 4B). Fisher’s LSD test indicated that the average droplet diameter varied significantly across all Φ values (Table S5). This was similar to that which has been observed in the literature, that decreasing Φ yields a significant change in droplet diameter [11]. Additionally, the initial flow instabilities at the T-junctions at the initiation of gravity-driven flow could result in inconsistent simultaneous droplet generation that is resolved after the system reaches equilibrium. Finally, droplet generation halted at Φ > 16 in the gravity-driven system when the shear stress applied by the oil flow prevented the aqueous phase from reaching the T-junction at the aqueous inlets, which was similar to single input studies described in the literature [43]. As such, Φ values greater than 16 were not investigated in this study for the gravity-driven flow system. Similarly, when Φ < 1, the water phase was found to infiltrate the oil inlet channel, resulting in the generation of massive droplets or wetting of the entire device.

### 3.3. Decreasing Φ Decreased the Droplet Generation Rate Using Gravity-Driven Flow

**Figure 5:**
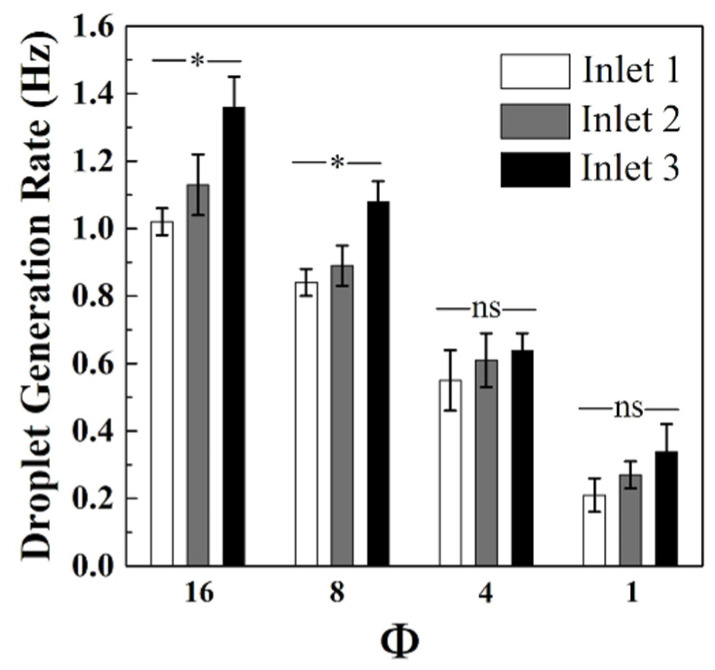
Droplet generation rate decreased as the oil-to-water ratio (Φ) value decreased in triple active gravity-driven experiments. The relationship between Φ and the generation rate was elucidated using all three active inlets. The mean droplet diameters from the three different inlets at the same oil-to-water ratio were significantly different for given Φ = 8 and 16. For each inlet, 30 droplets were measured, and the results are representative of three trials, with * denoting *p* > 0.05.

The droplet generation rates as a function of Φ were studied for gravity-induced droplet formation using the TA combination (Figure 5, Video S1). For all inlets, the generation rate was proportional to the change in Φ. For instance, in inlet 3, the generation rate shifted from 1.36 ± 0.07 to 0.34 ± 0.04 Hz as the Φ value changed from 16 to 1. These results were in accordance with previously developed models for T-junction geometries, where the viscous and interfacial tension force balance is still applicable to the prediction of droplet size and generation rate [41,44]. Taking both dispersed and continuous phase viscosities into account resulted in a fourth-order polynomial that can be solved for the droplet diameter [45]. As shown in Equation (S6), the droplet generation rate was inversely proportional to droplet diameter and directly proportional to the oil flow rate, similar to that which was observed in Figure 5. Performing ANOVA for different inlets at constant Φ values revealed that at oil-to-water flow rate ratios ≤4, the droplet generation rates were not significantly different (Table S6). In contrast, for Φ ≥ 8, the generation rates were different (specifically for inlet 3 when compared to inlets 1 and 2). However, this discrepancy in droplet generation did not translate to large differences in droplet distribution. For example, at Φ = 16 (the largest generation rate difference) the droplet distribution from inlets 1, 2, and 3 was 29.2, 32.5, and 38.3%, respectively. This means that 1 min of droplet generation is expected to produce 61.2 ± 2.5 (inlet 1), 68.1 ± 5.4 (inlet 2), and 81.4 ± 4.0 (inlet 3) droplets. At *p* = 0.05, Fisher’s LSD calculations revealed a significant difference in the mean droplet generation rates across the Φ values, acting as a meaningful generation rate control (Table S7). The generation rates of the developed triple-input, in-series T-junction droplet microfluidics can be enhanced by tuning other parameters such as oil and water channel width, oil viscosity, and the capillary number to generate well-defined droplets using a cost-effective flow initiation method, if necessary.

## 4. Conclusions

A multi-input microfluidic platform capable of simultaneous generation of monodisperse droplets has been developed. A gravity-induced flow approach was incorporated to simultaneously generate droplets from three separate inlets without the need for >3 syringe pumps. The fluid flow rates from each inlet were tuned based on a developed empirical model that accurately predicted the relationship between reservoir height and aqueous flow rate. Droplet generation rate and droplet size were adjusted through a series of experiments using gravity-driven flow, resulting in monodisperse droplets of diameters varying between 110.5 ± 4.6 µm and 180.6 ± 4.8 µm based on the oil-to-water ratio (Φ). Comparison studies between droplet diameter and droplet uniformity were performed, which found that decreased values of Φ led to increasing droplet diameters. Furthermore, it was shown that the droplet diameter significantly differs when lower Φ values are compared for all inlets but is not significantly different at larger Φ values. A comparison between the droplet generation rates demonstrated a different relationship with the Φ value, where decreasing the Φ resulted in a decrease in the generation rate in all inlets. In contrast to the droplet diameter measurements, the droplet generation rates significantly differ for higher Φ values but are not significantly different at lower Φ values. However, even at high Φ values, the discrepancy in generation rates does not considerably affect the inlet droplet distribution.

## Figures and Tables

**Table 1:** **Calculated model values for gravity-driven aqueous flow.** The predicted flow rate values were compared experimental values and the coefficient of variance (CV) was calculated for each inlet.

Inlet 1	Inlet 2	Inlet 3
A_1-2_ = 1.06	A_2-1_ = 0.8	A_3-1_ = 1.6
A_1-3_ = 1.06	A_2-3_ = 2.13	A_3-2_ = 2.93
A_1-2,3_ = 1.87	A_2-1,3_ = 2.40	A_3-1,2_ = 1.87
Predicted Q_1-2,3_ = 166.2	Predicted Q_2-1,3_ = 167.8	Predicted Q_1-2,3_ = 160
Measured Q_1-2,3_ = 152.6	Measured Q_1-2,3_ = 148.7	Measured Q_1-2,3_ = 147.6
CV = 8.9%	CV = 12.9%	CV = 8.4%

## Data Availability

The data that support the findings of this study are available within the article and its Supplementary Material.

## Supplementary Materials

The following are available online at https://www.mdpi.com/article/10.3390/mi12101211/s1, See Supplementary Material for information on development of an empirical model relating aqueous flow rate and reservoir height, quantification of gravity-driven flow rates as a function of height using different aqueous inlets configurations in the absence of oil flow, and statistical analysis of droplet diameter. Figure S1: Quantification of gravity-driven flow rates as a function of height using a single aqueous inlet in the absence of oil flow, Figure S2: Quantification of gravity-driven flow rates as a function of height using two aqueous inlets in the absence of oil flow, Figure S3: Quantification of gravity-driven flow rates as a function of height using three aqueous inlets the absence of oil flow, Video S1: Snapshot of simultaneous droplet generation in three active inlets using gravity-driven flow, Table S1: Evaluating the predictive capability of the model, Table S2: Statistical comparison of mean droplet diameter between different Φ values using active Inlets 1 and 2 experiment, Table S3: Statistical comparison of mean droplet diameter between different Φ values using active Inlets 1 and 3 experiment, Table S4: Statistical comparison of mean droplet diameter between different Φ values using active Inlets 2 and 3 experiment, Table S5: Statistical comparison of mean droplet diameter between different Φ values using active Inlets 1, 2, and 3 experiment, Table S6: ANOVA on the mean droplet generation rate for all three active aqueous inlets for a specific oil-to-water ratio (Φ) value using gravity driven flow., Table S7: Statistical comparison of mean droplet generation rate between different Φ values using active Inlets 1 and 3 experiment.
