# Supplementary material for: Simultaneous Droplet Generation with In-Series Droplet T-Junctions Induced by Gravity-Induced Flow
Source: Micromachines (Basel). 2021 Oct 4;12(10):1211. doi: 10.3390/mi12101211 (PMC8540845; doi:10.3390/mi12101211)
Supplement: Supplementary file 1 [file micromachines-12-01211-s001.zip › micromachines-1392478-supplementary materials/micromachines-1392478-Supplementary.zip/micromachines-1392478-supple-proof done.pdf]

Simultaneous Droplet Generation with In-series Droplet T-Junctions Induced by Gravity-induced Flow

Khashayar R. Bajgiran, Alejandro S. Cordova, Riad Elkhanoufi, James A. Dorman*, and Adam T. Melvin*

Cain Department of Chemical Engineering, Louisiana State University, Baton Rouge, LA, 70803

Development of an Empirical Model Relating Aqueous Flow Rate and Reservoir Height

For the first set of experiments involving a single active aqueous inlet in the absence of oil flow, the Hagen-Poiseuille equation[20] was used to describe the relationship between the inlet flow rate and the pressure drop:

$$\boldsymbol{Q = \frac{\Delta P H^3 w}{12 v L}} \qquad \textbf{Equation S1}$$

where Q was the volumetric flow rate, ΔP was the pressure drop, h was the channel height, w was the channel width, v was the fluid velocity, and L was the channel length. The flow rate of each inlet was measured by monitoring the changes in liquid height in the respective reservoirs over time. These measurements were then verified by comparing the total dispensed volume of all inlets with the amount of liquid collected from the outlet, where the values were almost identical.

Using the empirical findings from aqueous flow-only experiments, including single, double, and triple active inlets, a mathematical model was developed to predict the approximate flow rate of each inlet at a given reservoir height, which is presented below

$$\boldsymbol{Q_{i-j,k} = \frac{1}{3}\left(A_{i-j} + A_{i-k} + A_{i-j,k}\right)\Delta H_{i-j,k}} \qquad \textbf{Equation S2}$$

$$\boldsymbol{A_{i-j} = \frac{Q_i - Q_{i-j}}{\Delta H_{i-j}}} \qquad \textbf{Equation S3}$$

$$\boldsymbol{A_{i-j,k}} = \frac{\boldsymbol{Q_i} - \boldsymbol{Q'_{i-j,k}}}{\Delta \boldsymbol{H'_{i-j,k}}}$$ **Equation S4**

where $Q_{i\text{-}j,k}$ (µL/h) was the flow rate of inlet i when all three inlets were active and connected to different (optimized) reservoir heights, $A_{i\text{-}j}$ (µL/(h-cm)) was a constant parameter that was calculated based on the difference of the inlet i flow rate when it was the only active inlet (Q_i) and inlet i flow rate when both inlets i and j were active ($Q_{i\text{-}j}$) divided by the height of inlet i reservoir when inlets i and j were active and their reservoir were at the same height ($\Delta H_{i\text{-}j}$ = 125 cm). Similarly $A_{i\text{-}j,k}$ (µL/(h-cm)) was a constant parameter calculated based on the difference of the inlet i flow rate when it was the only active inlet (Q_i) and inlet i flow rate when all three inlets i, j, k were active ($Q_{i\text{-}j,k}$) divided by the height of inlet i reservoir when all three inlets i, j, k were active and their reservoirs were at the same height ($\Delta H'_{i\text{-}j,k}$ = 125 cm). $\Delta H_{i\text{-}j,k}$ was the height of inlet i when all three inlets i, j, k were active and their reservoirs were at different (optimized) heights.

Optimization of External Aqueous Reservoir Height to Generate Uniform Droplets by Gravity-Driven Flow

To characterize the gravity-induced flow inside the device, the flow rates of different active aqueous inlets were measured in the absence of oil flow using reservoirs mounted on a custom-built ring stand at the same height (125 cm). In this study, the active inlet was defined as an inlet that is punched, connected to a reservoir, and with aqueous flow running through the device. Inactive inlets were not punched or connected to a reservoir. Single, double, and triple active inlets were evaluated to determine the effects of inlet number/position on the flow rate of the inlets at similar reservoirs' heights (125 cm). An empirical model was developed based on the findings from these experiments that related the inlet number/reservoir height to their respective flow rates (see supporting information). Using this model, the heights of each inlet reservoir were adjusted to yield similar flow rates when all inlets were active, with inlet 1 at 125 cm, inlet 2 at 95 cm, and

inlet 3 at 75 cm. In all experiments, 15 mL conical tubes (VWR) were filled with 12 mL of dH_2O and were connected to their respective inlets using Tygon tubing (with an inner diameter of 0.56 mm, Cole Palmer). Tubing was sealed at the connection point using cured PDMS to prevent reservoir leakage. The outlet was connected to a 50 mL conical tube (VWR), positioned at the same height as the device) using Tygon tubing. The experiments were run over 90 min periods, and the changes in inlet reservoir volume were recorded every 15 min. In each experiment, the volume of collected dH_2O from the outlet was compared to the sum of the volume changes of the active inlets to ensure that no leakage has happened and no volume difference was found.

Investigation on the Effect of Φ on Droplet Diameter and Generation Rate Using Gravity-Driven Flow

Using the optimized heights, aqueous droplets were generated from multiple combinations of aqueous inlets in the presence of oil flow that was initiated using a syringe pump. The oil phase used in all experiments was Novec 7500 oil supplemented with 2 (w/w) % Neat 008 fluorosurfactant (Ran Biotechnologies). For all active inlet combinations (Table 2), fluorescein isothiocyanate (FITC, 100 µM) and Rhodamine 6G (100 µM) were used in inlets 2 and 3 to identify the droplets using fluorescent microscopy. Inlet 1 only contained dH_2O, which resulted in non-fluorescent droplets. The experiments were performed by varying the oil inlet flow rates (30 to 300 µL/hr) at a fixed aqueous inlet flow rate (25 µL/hr) to alter the oil-to-water flow ratio (Φ) defined as

$$\boldsymbol{\Phi} = \frac{\boldsymbol{Q_o}}{\boldsymbol{Q_w}} \qquad \textbf{Equation S5}$$

where Q_o and Q_w were the volumetric oil and water flow rates, respectively. For each Φ, the droplets were imaged primarily in the opening of the main channel (Figure 1C). The device was mounted on a fluorescent DMi8 inverted microscope (Leica microsystems) outfitted with a 10×

objective (Leica HC PL FL L, 0.4× correction) under brightfield for all experiments. Images were acquired using the digital CMOS camera C11440 (Hamamatsu Photonics K.K.) with a fixed exposure time of 100 ms for the FITC and rhodamine filters and 25 ms for brightfield. Image acquisition was controlled using the Leica Application Suite software (LAS X), where all images were recorded using the same parameters. The following excitation/emission filters (Chroma Tech. Corp) were used to image the device: FITC (λ_{ex} 440–520 nm and λ_{em} 497–557 nm); rhodamine (λ_{ex} 536–556 nm and λ_{em} 545–625 nm).

The relation between the Φ and droplet generation rates was investigated by a series of gravity-driven droplet formation experiments with triple active inlets. The results were compared to previously developed models for T-junction geometry developed by Husny and Cooper-White.[46] According to this model, the droplet generation rate can be obtained based on mass conservation using the equation below

$$\textbf{Generation Rate} = \frac{6Q_o}{\pi D^3} \qquad \textbf{Equation S6}$$

where Q_o and D were the volumetric oil flow rate and the droplet diameter, respectively.

Data processing and statistical analysis

For all experiments studying the droplet size, a manual line scan was drawn across individual droplets to measure their diameters using LAS X software. For each experiment, the mean diameter was measured for at least 30 droplets from each aqueous inlet for the four Φ values. The mean diameters were further analyzed using an ANOVA to determine the relationship between the droplet size, Φ, and the aqueous inlet number and position using Origin software. ANOVA was performed to assess the statistical significance of the mean droplet size at particular Φ values and across different Φ values in gravity-driven experiments. The threshold (p-value) for all experiments was set to 0.05. The mean droplet size for all combinations of Φ was analyzed

using Fisher's least significant difference (LSD) test. Droplet generation rates were obtained by monitoring droplet generation at each T-junction using a recorded video for a total of 45 s with a capture rate of 25 frames/s using manual counting. The measurements were performed on each Φ value for the three active inlet droplet generation experiments in gravity-driven flow. Similar ANOVA and Fisher LSD tests were performed on droplet generation rate results.

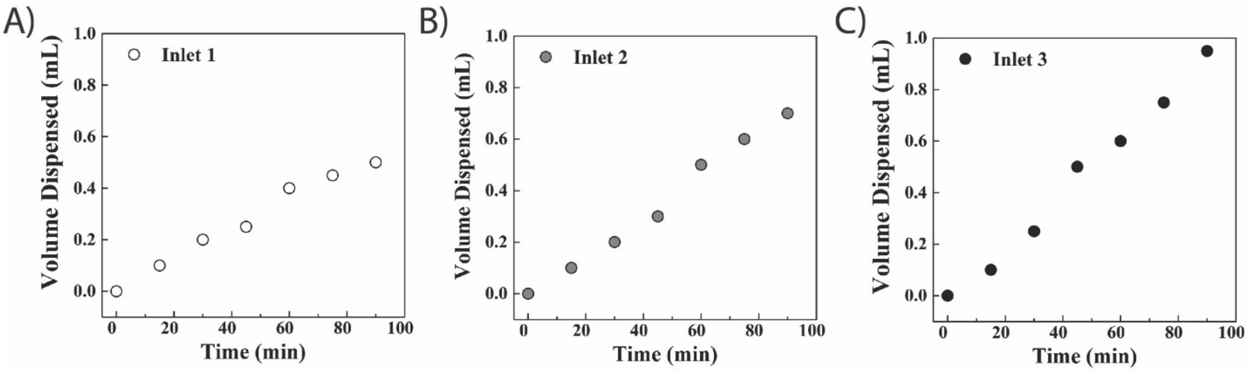

Figure 1. Quantification of gravity-driven flow rates as a function of height using a single aqueous inlet in the absence of oil flow. The average inlet flow rate was measured for each inlet by dividing the dispensed reservoir volume over time, for a single active aqueous inlet 1(A, 333.3 µL/h), 2 (B, 466.6 µL/h), or 3 (C, 633.3 µL/h). All inlet reservoirs were positioned 125 cm above the device.

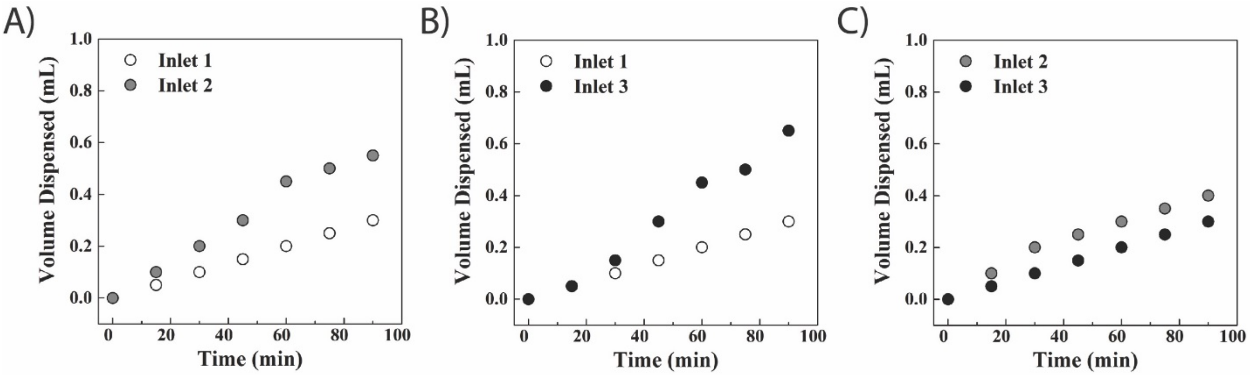

Figure S2. Quantification of gravity-driven flow rates as a function of height using two aqueous inlets in the absence of oil flow. The average inlet flow rate was measured for each inlet by dividing the dispensed reservoir volume over time, for two active aqueous inlets 1 and 2 (A, 200 and 366.7 µL/h, respectively), 1 and 3 (B, 200 and 433.3 µL/h, respectively), or 2 and 3 (C, 200 and 266.7 µL/h, respectively). All inlet reservoirs were positioned 125 cm above the device in all experiments.

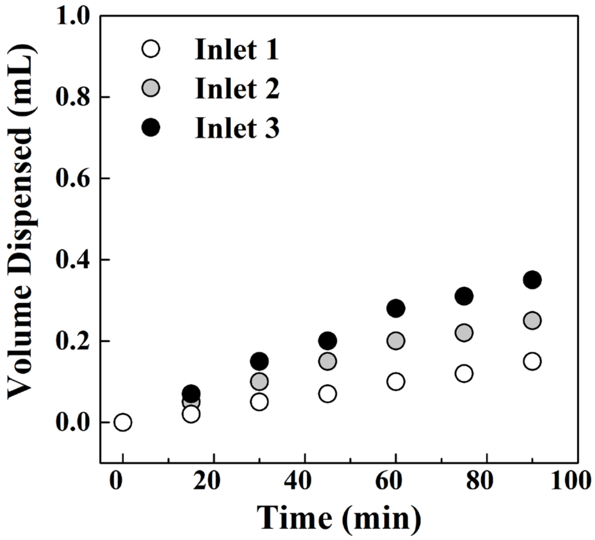

Figure S3. Quantification of gravity-driven flow rates as a function of height using three aqueous inlets the absence of oil flow. The average inlet flow rate was measured for each inlet by dividing the dispensed reservoir volume over time, for three active aqueous inlets 1 (100 µL/h), 2 (166.7 µL/h), and 3 (233.3 µL/h). All inlet reservoirs were positioned 125 cm above the device in all experiments.

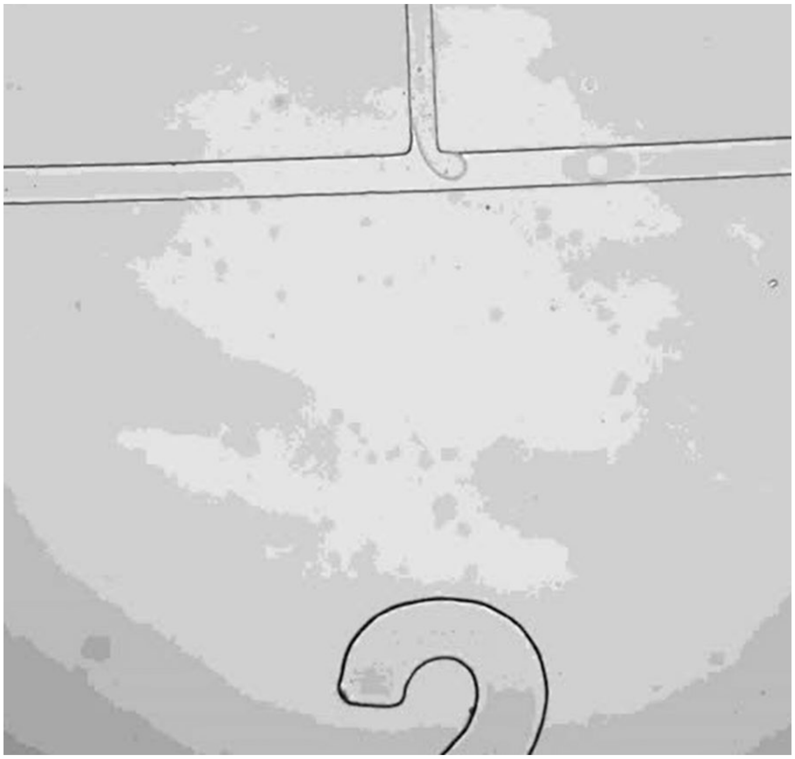

Video S1. Snapshot of simultaneous droplet generation in three active inlets using gravity-driven flow. The droplets were generated at $\Phi = 4$. The video frame rate is 15 frame/s.

Table S1. Evaluating the predictive capability of the model. The height of each inlet reservoir was adjusted to yield 100 μL/h flow rate according to the model. The set flow rate value was compared to those of the experimentally measured, and the coefficient of variance (CV) was calculated for each inlet.

**Gravity Flow Rate in the absence of Oil**			
**Inlet**	**Inlet 1 (75 cm)**	**Inlet 2 (55 cm)**	**Inlet 3 (47 cm)**
Desired Flow Rate (μL/h)	100	100	100
Measured Flow Rate (μL/h)	110.4	104.4	105.9
CV (%)	10.4	4.4	5.9

Table S2. Statistical comparison of mean droplet diameter between different *Φ* values using active Inlets 1 and 2 experiment. The averaged mean droplet diameters were compared across different oil-to-water flow rate ratio (*Φ*) values using a Fisher LSD. The calculations revealed that the droplet diameter are significantly different between different (*Φ*) values.

Φ	MeanDiff	SEM	t Value	Prob	α	Diff/No Diff
16 vs. 8	7.24	2.01	3.61	3.72E-04	0.05	Difference
16 vs. 4	15.51	2.01	7.73	3.09E-13	0.05	Difference
8 vs. 4	8.26	2.01	4.12	5.27E-05	0.05	Difference
16 vs. 1	60.58	2.01	30.20	5.01E-83	0.05	Difference
8 vs. 1	53.34	2.01	26.59	6.28E-73	0.05	Difference
4 vs. 1	45.07	2.01	22.47	1.51E-60	0.05	Difference

Table S3. Statistical comparison of mean droplet diameter between different *Φ* values using active Inlets 1 and 3 experiment. The averaged mean droplet diameters were compared across different oil-to-water flow rate ratio (*Φ*) values using a Fisher LSD. The calculations revealed that the droplet diameter are significantly different between different (*Φ*) values.

Φ	MeanDiff	SEM	t Value	Prob	α	Diff/No Diff
16 vs. 8	7.81	3.45	2.26	0.025	0.05	Difference
16 vs. 4	17.02	3.45	4.93	1.54E-06	0.05	Difference
8 vs. 4	9.21	3.45	2.67	0.00811	0.05	Difference
16 vs. 1	56.06	3.45	16.24	2.49E-40	0.05	Difference
8 vs. 1	48.25	3.45	13.98	9.24E-33	0.05	Difference
4 vs. 1	39.04	3.45	11.31	5.52E-24	0.05	Difference

Table S4. Statistical comparison of mean droplet diameter between different *Φ* values using active Inlets 2 and 3 experiment. The averaged mean droplet diameters were compared across different oil-to-water flow rate ratio (*Φ*) values using a Fisher LSD. The calculations revealed that the droplet diameter are significantly different between different (*Φ*) values.

Φ	**MeanDiff**	**SEM**	**t-Value**	**Prob**	**α**	**Diff/No Diff**
16 vs. 8	10.39	2.31	4.50	1.07E-05	0.05	Difference
16 vs. 4	17.40	2.31	7.53	1.06E-12	0.05	Difference
8 vs. 4	7.00	2.31	3.03	0.0027	0.05	Difference
16 vs. 1	58.23	2.31	25.21	6.79E-69	0.05	Difference
8 vs. 1	47.84	2.31	20.71	5.34E-55	0.05	Difference
4 vs. 1	40.83	2.31	17.68	4.13E-45	0.05	Difference

Table S5. Statistical comparison of mean droplet diameter between different *Φ* values using active Inlets 1, 2, and 3 experiment. The averaged mean droplet diameters were compared across different oil-to-water flow rate ratio (*Φ*) values using a Fisher LSD. The calculations revealed that the droplet diameter are significantly different between different (*Φ*) values.

Φ	**MeanDiff**	**SEM**	**t Value**	**Prob**	**α**	**Diff/No Diff**
16 vs. 8	5.37	2.60	2.06	0.04	0.05	Difference
16 vs. 4	15.65	2.60	6.01	4.65E-09	0.05	Difference
8 vs. 4	10.28	2.60	3.95	9.54E-05	0.05	Difference
16 vs. 1	63.68	2.60	24.45	3.53E-78	0.05	Difference
8 vs. 1	58.31	2.60	22.387	6.48E-70	0.05	Difference
4 vs. 1	48.03	2.60	18.44	8.87E-54	0.05	Difference

Table S6. ANOVA on the mean droplet generation rate for all three active aqueous inlets for a specific oil-to-water ratio (*Φ*) value using gravity driven flow. The ANOVA revealed that at $\Phi \leq 4$ the droplet generation rates are not significantly different. Conversely, for $\Phi \geq 8$, the generation rates are different. p-value was set to 0.05 for all tests.

Φ	Null Hypothesis	F-value	Pr > F	Accept/Reject Null
1	Mean1=Mean2=Mean3	4.57	0.06	Accept
4	Mean1=Mean2=Mean3	0.61	0.57	Accept
8	Mean1=Mean2=Mean3	11.44	0.01	Reject
16	Mean1=Mean2=Mean3	12.27	0.01	Reject

Table S7. Statistical comparison of mean droplet generation rate between different *Φ* values using active Inlets 1 and 3 experiment. The averaged mean droplet diameters were compared across different oil-to-water flow rate ratio (*Φ*) values using a Fisher LSD. The averaged mean droplet generation rates were compared across different oil-to-water flow rate ratio (*Φ*) values. The calculations revealed that the droplet generation rates are significantly different between different (*Φ*) values.

Φ	MeanDiff	SEM	t Value	Prob	α	Diff/No Diff
16 vs. 8	0.23	0.06	-4.18	2.11E-04	0.05	Difference
16 vs. 4	0.57	0.06	-10.18	1.46E-11	0.05	Difference
8 vs. 4	0.34	0.06	-6.00	1.08E-06	0.05	Difference
16 vs. 1	0.89	0.06	-15.93	9.11E-17	0.05	Difference
8 vs. 1	0.66	0.06	-11.75	3.84E-13	0.05	Difference
4 vs. 1	0.32	0.06	-5.74	2.28E-06	0.05	Difference
